# Exercise training and post-operative prognosis after coronary intervention

**DOI:** 10.1186/1471-2482-13-S1-A33

**Published:** 2013-09-16

**Authors:** Gennaro Pagano, Dario Leosco, Nicola Ferrara, Nicola Rocco, Corrado Rispoli, Loredana Iannone, Serena Testa, Antonello Accurso, Rita Compagna, Bruno Amato

**Affiliations:** 1 Department of Translational Medical Sciences, Federico II University of Naples, Naples, Italy; 2 “Salvatore Maugeri” Foundation, IRCCS Scientific Institute of Telese Terme, Benevento, Italy; 3 Department of Biomedical, Surgical and Dental Sciences, University of Milan, Milan, Italy; 4 Department of General Surgery - ASL NA1, Cardinale Ascalesi Hospital, Naples, Italy; 5 Department of General Surgery, Fatebenefratelli Hospital, Benevento, Italy; 6 Department of General, Geriatric, Oncologic Surgery and Advanced Technologies, Federico II University of Naples, Naples, Italy

## 

Aging is worldwide recognized as a dominant risk factor for most forms of cardiovascular disease [1-3]. However, mechanisms by which it exerts its role and determines poor outcome have been only partially clarified. Numerous evidence indicate that aging is associated with alteration of several mechanisms whose integrity confers protective action on the heart and vasculature [5-9]. Autonomic status derangement, diminished efficacy of ischemic preconditioning, impaired angiogenic responses after ischemic injury, increased oxidative stress, and abnormal left ventricular remodelling after myocardial infarction are all putative mechanisms potentially involved in the vulnerability of cardiovascular system occurring with aging. Interestingly, many of the alterations that take place in the aged heart and vasculature are very similar to those observed in pathologic conditions, such as heart failure (HF), and, most importantly, are at least in part revertible. Exercise training plays a pivotal role in primary and secondary prevention of cardiovascular disease, in counteracting the age-related deterioration of some mechanisms that are crucially involved in the homeostasis of cardiovascular system and that may condition the outcome of cardiovascular disease in the elderly. Moreover, it is associated to reduction of the post-operative atrial fibrillation with reduction of the number of prescribed drugs [10]. Preconditioning represent the strongest form of in vivo protection against myocardial ischemic injury, consisting in brief episodes of myocardial ischemia able to reduce cellular damage subsequent to a more prolonged ischemic injury. Remote preconditioning is the phenomenon by which ischemia in one region of the heart causes protection in a remote region of the heart itself or of another organ, thus suggesting that circulating factors or a neural reflex triggers protection in the remote region. Aging is associated with higher rates of morbidity after coronary intervention and a progressive loss in efficacy of ischemic preconditioning with age has been indicated as a potential mechanism explaining the worse prognosis in the elderly. A diminished norepinephrine release in response to transient ischemic stress has been indicated having role in the loss of preconditioning protection occurring with age. Preinfarction angina is considered one of the strongest clinical equivalent of ischemic preconditioning being associated with both reduction in infarct size and prevention of left ventricular dysfunction. Evidences demonstrate the loss of the protective effect of preinfarction angina with age, resulting in a no more protective effect on both early and late mortality in the elderly after acute cardiac events.

In adult and elderly humans, high levels of physical activity preserve the protective role of preinfarction angina against in-hospital mortality and cardiogenic shock after myocardial infarction. More recent data indicate that higher levels of physical activity performed before primary coronary angioplasty may independently predict the reduction of early and late cardiac mortality in older infarcted patients [11]. Accordingly, it has been reported a robust association between physical activity habits of elderly participants prior to coronary artery bypass grafting and survival free from both all-cause and cardiac death, [12]. Interestingly, in these studies, the greatest benefits of exercise on survival were observed in the more sedentary patient groups, thus strongly supporting the importance of implementation of physical activity levels in the elderly also as secondary prevention strategy.
